# The implementation of conscious sedation by dental professionals in Egypt: an analytical cross-sectional study

**DOI:** 10.1038/s41598-024-66834-z

**Published:** 2024-07-17

**Authors:** Mohamed Taha Elfezary, Mohamed Elsayed Moteea, Mohammed Said Abu Samadah, Ahmed S Waly

**Affiliations:** Department of Pediatric Dentistry, Faculty of Dentistry, Al-Azhar University, Assiut, Egypt

**Keywords:** Egypt, Conscious sedation, Pediatric dentistry, Anxiety, Anesthesiologist, Health care, Dentistry, Dental treatments

## Abstract

Conscious sedation is widely considered one of the techniques most commonly used to manage anxiety in both children and adults during dental procedures. The application of procedural sedation exhibits considerable variation globally. The purpose of the study was to determine the prevalence of conscious sedation in the current situation in the dental healthcare sector in Egypt and to identify the factors influencing it. An online cross-sectional analytical survey, consisting of nine multiple choice questions, was distributed through the contact lists of national dentists and social media platforms. 163 respondents completed the survey. Regarding the use of conscious sedation, only 25 (15.3%),95% confidence interval (10–21) utilized it. The percentage of participants who used conscious sedation was higher among dentists practicing pediatric dental specialists (n = 19, 76%). Academic qualification correlates significantly with the practice of conscious sedation (*P* = 0.002), but this was not reflected in multiple logistic regression. Indeed, while conscious sedation is part of the dental study curriculum in Egypt, its application rate is relatively low compared to other countries. This discrepancy could be attributed to a variety of factors, including resource availability, lack of training, reflecting the need to develop strategies to improve the implementation of conscious sedation in Egyptian dental practices.

## Introduction

The concern about dental procedures is a widely recognized problem. Dental anxiety, characterized by feelings of fear or anxiety when faced with the prospect of dental treatment, has been identified as one of the main reasons why people avoid dental care^1^. A recent comprehensive review revealed that anxiety about dentistry and elevated dental anxiety are quite prevalent worldwide^2^. In Egypt, a study found that 46.5% of individuals reported experiencing dental anxiety^3^.

Effective management of patient behavior is essential for successful dental treatment^4^. There are various techniques available to manage patient behavior. Conscious sedation is a notable method and is widely considered one of the most frequently used techniques to manage anxiety in both children and adults. during dental procedures^5^. The American Academy of Pediatric Dentistry (AAPD) defines Conscious sedation is a medically supervised condition of decreased awareness that allows the patient to preserve his defensive reflexes, maintain an open airway, and respond appropriately to physical stimuli and verbal commands^6^.

Conscious sedation serves as an exceptional tool for handling patients with dental apprehension who require dental procedures. Studies have indicated that the technique is safe and effective when administered orally, intravenously, or by inhalation^7^. The delivery of procedural sedation varies worldwide. In some regions, anesthesiologists are the exclusive providers of this treatment in surgical entities (for example, the majority of Europe), while in others, a few trained non-anesthesiologists provide sedation in specific situations and places (for example, the United Kingdom [UK]). The publication of A Conscious Decision in July 2000, a report by a group chaired by the Chief Medical Officer and Chief Dental Officer of England (subsequently accepted by the British Government); Additionally, it reinforced the necessity for conscious sedation as a technique for managing anxiety^7^. This report advocated for suitable training at both the undergraduate and postgraduate levels in sedation methods, a perspective supported by the Royal Colleges, the General Dental Council, and the American Dental Association^8,9^.

In several other nations, several nonmedical practitioners administer sedation in various environments (for example, the United States and Canada)^10^. In Egypt, Anesthesiology is a discipline limited to doctors, following a 6-year medical undergraduate course, physicians can apply for a 3-year anaesthesiology residency program. The competencies of practitioners and standards of practice are frequently debated topics in the field of procedural sedation^10,11^. According to the Joint Commission on Accreditation of Healthcare Organizations, a qualified hospital sedation provider is one who has 'at a minimum had competency-based education, training, and experience' in evaluating patients, performing moderate sedation, and rescuing patients who fall into a deeper level than desired sedation^12^.

It has been suggested that a shortage of anesthesiologists is one factor that limits the expansion of sedation services^11,13,14^. There is a wide range of dental training experiences to prepare dentists to provide dental sedation^15^. With the increasing demand for sedation in dentistry, it is necessary to clarify the professional roles in providing this type of sedation^16^. In Egypt, there is a significant information gap on the prevalence of conscious sedation practices among dentists. This survey represents the first study to determine the current application of conscious sedation in dental treatments in Egypt, as well as to identify the factors that influence its use.

## Methods

### The aim of study

Assess and understand current practices and attitudes of dentists in Egypt toward conscious sedation.

### Study design

A web-based online research survey was conducted from the start of October 2023 to the middle of October 2023. The study was approved by the Research Ethic Committee of the Faculty of Dental Medicine, Al-Azhar University, Assiut (AUAREC202300009-17). The research adheres to the Helsinki Declaration protocols All participants provided their informed consent and were thoroughly informed about the objectives and advantages of the study.

### Study participants and sample size

The sample size was calculated to be 120 based on similar studies^17^ and 8.5% was hypothesized to be the frequency of outcome factors in the population and (95% confidence level, 5% error margin) by power analysis (OpenEpi Sample Size Calculator). Averaging 20% of excluded responses, a total sample size of 144 participants is needed to achieve the objectives of this study.

### Recruitment of study participants

Dentists were invited to participate in the survey through national dentist contact directories and individual social media pages that included a link to the online survey. The digital questionnaire was conducted via Google e-forms, and each dentist was allowed to submit just one response.

### Data collection tool

An online questionnaire, consisting of nine questions, was developed. To ensure clarity, comprehensibility, and minimize ambiguity in responses, the questionnaire was validated based on a similar previous study^7^ and initially evaluated in a group of three experienced dentists. The design of the questionnaire was intended to provide comprehensive response options. An introductory page preceded the survey providing information on the objectives of the survey, details about the research team, and an invitation to participate in this anonymous survey. Consent was implied through participation in the survey, which made it an opt-in survey.

The questionnaire contained a series of multiple-choice questions that covered various topics

- *Demographics*: This section collected information about the respondent’s gender, work experience, specialty status, and academic qualifications.
- *Sedation in Practice*: This section asked questions about:
  - the use of conscious sedation in the practice of the respondent.
  - whether they use conscious sedation, whether they administer it independently or with an anesthesiologist.
  - whether they are trained and certified in providing conscious sedation.
- *No Sedation in Practice*: This section asked respondents:
  - who do not use conscious sedation about their reasons for not using it.
  - whether they would consider further postgraduate training in sedation techniques.

### Statistical analysis

Data were tabulated, coded and statistically described in terms of frequencies (number of cases) and percentages, prevalence and 95% confidence interval were calculated, while logistic regression was used to identify the association between baseline characteristics of the participants and the use of conscious sedation. Statistical analysis was performed using IBM SPSS 28 (Statistical Package for Social Sciences).

### Ethics approval and consent

The study was approved by The Research Ethic Committee, Faculty of Dental Medicine, Al-Azhar University, Assiut (AUAREC202300009-17). All participants provided their informed consent, and they were thoroughly briefed about the study’s objectives and advantages.

## Results

163 Egyptians completed the survey. However, this resulted in a total of 163 participants achieving the power of the study.

Gender: The survey included more female (52.1%) than male (47.9%). Experience Level: Most of the participants had < 6 years of experience (44.8%), followed by those with 6 -15 years of experience (42.9%). Participants with 16–25 years and > 25 years of experience were fewer, Academic Qualifications: The majority had a bachelor’s degree (47.9%), followed by holders of master's degrees (33.1%). Participants with a Doctorate degree and those with a diploma were the smallest groups. Specializations: Pediatric dentistry was the most common specialty (56.4%), followed by general practice (34.4%). Other specializations had much lower frequencies (Table 1).

**Table 1:** Frequency and bivariate analysis of practice of conscious sedation with baseline characteristics.

	Do you utilize conscious sedation?		Total Count %	P. V
	No	Yes		
	Count %	Count %		
Gender				
Male	62 (79.5%)	16 (20.5%)	78 (47.9%)	.079
Female	76 (89.4%)	9 (10.6%)	85 (52.1%)	
Work experience				
< 6	66 (90.4%)	7 (9.6%)	73 (44.8%)	.131
6–15	54 (77.1%)	16 (22.9%)	70 (42.9%)	
16–25	13 (92.9%)	1 (7.1%)	14 (8.6%)	
> 25	5 (83.3%)	1 (16.7%)	6 (3.7%)	
Specialty				
GP	53 (94.6%)	3 (5.4%)	56 (34.4%)	.241
Periodontology	2 (100.0%)	0 (0.0%)	2 (1.2%)	
Pediatric Dentistry	73 (79.3%)	19 (20.7%)	92 (56.4%)	
Restorative	3 (75.0%)	1 (25.0%)	4 (2.5%)	
Orthodontic	1 (100.0%)	0 (0.0%)	1 (0.6%)	
Maxillofacial Surgery	2 (66.7%)	1 (33.3%)	3 (1.8%)	
Others	4 (80.0%)	1 (20.0%)	5 (3%)	
Academic Qualification				
Bachelor's Degree	72 (92.3%)	6 (7.7%)	78 (47.9%)	.002
Diploma	15 (100.0%)	0 (0.0%)	15 (9.2%)	
Master’s Degree	38 (70.4%)	16 (29.6%)	54 (33.1%)	
Doctoral Degree	13 (81.3%)	3 (18.8%)	16 (9.8%)	

Regarding the use of conscious sedation, 25 individuals accounted for 15.3%, while those who did not use it accounted for 138 individuals at a percentage of 84.7% and 95%CI (10–21). Of the twenty-five individuals who used conscious sedation, twenty-two individuals, or 88%, practiced it under the supervision of an anesthesiologist. The remaining three individuals, or 12%, practiced it independently, one of them being a trained professional. However, the other two individuals did not receive any training.

The percentage of participants using conscious sedation), The higher among individuals with a master’s degree (n = 16, 64%), dentists practicing pediatric dental specialists (n = 19, 76%) and those with 6–15 years of work experience (n = 16, 64%),The application of conscious sedation demonstrated a significant correlation with the academic qualification of the participants, as evidenced by the bivariate analysis (*P* = 0.002) (Table 1).To account for possible confounder variables, a multiple logistic regression was used. However, this adjustment did not yield statistically significant results (Table 2).

**Table 2:** Multiple logistic regression predicting conscious sedation practice.

	Sig	Exp(B)	95% C.I.for EXP(B)	
			Lower	Upper
Gender				
(Female)	.063	.375	.133	1.056
Male	Reference			
Work Experience	.677			
< 6	Reference			
(6–15)	.667	.725	.168	3.134
(16–25)	.228	.185	.012	2.875
(> 25)	.655	.524	.031	8.922
Specialty	.906			
GP	Reference			
(Periodontology)	.999	.000	.000	
(Pediatric Dentistry)	.692	1.499	.203	11.060
(Restorative)	.186	6.307	.411	96.735
(Orthodontic)	1.000	.000	.000	
(Maxillofacial Surgery)	.457	3.284	.143	75.277
(Others)	.603	2.184	.115	41.496
Academic Qualification	.277			
Bachelor's degree	Reference			
(Diploma)	.999	.000	.000	
(Master's degree)	.051	6.193	.996	38.528
(Doctoral degree)	.218	4.363	.418	45.515

The main barriers to the use of conscious sedation included availability (24.6%, n = 34), lack of training (15.2%, n = 21) and preference for general anesthesia (7.2%, n = 10). Other reported reasons for barriers to the use of conscious sedation included the preference of the patient / parent and combined barriers (Table 3).

**Table 3:** Barriers of conscious sedation practice.

Barriers	Count %
Availability	34 (24.6%)
Lack of training or expertise	21 (15.2%)
I prefer to undergo general anesthesia	10 (7.2%)
Patient/parent preference	1 (0.7%)
Combined barriers	72 (52.1%)

When asked about their interest in training, among those who do not currently practice conscious sedation, 124 (89.8%) expressed interest in training. On the contrary, 14 (10.1%) indicated a lack of interest in the training.

## Discussion

Delays in dental treatment can lead to numerous complications due to stress and fear of pain. This is particularly true for children and people with special needs. The objective goes beyond simply treating dental diseases; it also aims to foster a positive attitude toward future dental treatments. Conscious sedation serves as a crucial tool in dentistry for the management of such patients, especially when addressing a limited number of dental issues and emergencies that cannot be treated with local or general anesthesia due to potential risks, including death.

During the past few decades, there has been an increase in the number of minor diagnostic and surgical procedures performed on pediatric patients outside of the traditional operating room setting. This transition, along with an increased understanding of the need for pain relief and anxiety reduction, has markedly increased the need for sedation in dental practices^6^.

The findings of our research offer a fair depiction of the prevailing views on the application of conscious sedation in Egypt. The feedback is an authentic mirror of the practical utilization and necessity of sedation.

The primary finding of our study is that only 15.3% (n = 25) of 163 dentists use conscious sedation in dental medicine in Egypt, which is a notably low percentage. The primary reasons for this low percentage include the lack of availability, training, and qualifications to administer this type of sedation, compounded by the ban on nitrous oxide in Egypt. Additionally, government restrictions on dispensing such drugs due to their potential for misuse are difficult to enforce. This is consistent with the findings of Al-Shayyab et al. and Chadwick BL, who reported that only 8.5% in Jordan and 12.1% in Wales of respondents, respectively, perform some form of conscious sedation in their practice^17,18^.

In contrast, Foley J. and Whiston S reported much higher frequencies-49% and 42%, Scotland and northern England, respectively-over twenty years ago^7,19^.

Our study found that pediatric dentistry is the most common specialty that uses conscious sedation, accounting for 76% (n = 19) of the cases. This finding can be attributed to the higher prevalence of noncooperation among children with dental practitioners.

Morse Z. reported that techniques to manage both anxiety and pain are often crucial in the treatment of special patients, young children, and those with mental and physical challenges. In this group of patients, sedation and analgesia are essential^20^. However, Al-Shayyab et al. reported that only 17.8% of dentists in the pediatric dentistry category use conscious sedation^17^.

In our study, we found that 88% (n = 22) of the physicians administer conscious sedation under the supervision of an anesthesiologist. This highlights the need for dentists to be qualified and trained in administering this type of sedation. Many anesthesiologists in Central West Brazil lack confidence in dentists’ ability to administer dental sedation. The responsibilities of dentists and physicians in this region need to be clarified to benefit the population^21^.

As the need for conscious sedation in dentistry escalates, it becomes unfeasible for it to be administered exclusively by anesthesiologists. Consequently, it is crucial to obtain the support of our medical peers to provide conscious sedation and increase public trust in our care provision^16^.

Regarding training aspirations, we found that 89.9% of practitioners expressed a desire to train in conscious sedation. This underscores its importance and the lack of available training courses for dentists, government or private. Currently, there are no recommendations from Egypt's health authorities on this matter.

Historically, sedation in nonhospital settings (eg, private physicians or dental offices) has been associated with an increased incidence of ‘failure to rescue’ from adverse events due to a lack of immediately available support^6^. Therefore, training and qualification are essential, and Egypt's health authorities and Dental Associations should organize courses and training programs on this topic, as it can benefit society.

### Study limitations

Indeed, this research, despite its limitations, pioneers the investigation of the use of conscious sedation in Egypt. It is crucial to acknowledge certain limitations. Primarily, the study was carried out with a limited sample size and conducted online. Although this approach is cost-effective and efficient, it may inadvertently exclude individuals who lack internet access or those who are not inclined to participate in online surveys.

Furthermore, the study’s focus was exclusively on Egyptian dentists, thus restricting the generalizability of the findings to other geographic locations or demographics. Despite these limitations, this research significantly contributes to the understanding of conscious sedation practices in Egypt. It sets the groundwork for future studies and has the potential to influence policy and practice within the region.

## Conclusions

1. The use of conscious sedation techniques to treat patients with dental anxiety is low among dental practitioners in Egypt.
2. Pediatric dentistry provides most of the sedation services in dental settings in Egypt.
3. The primary factors preventing the implementation of conscious sedation are inadequate training and limited accessibility.
4. Most believed that sedation was required within their own practice.
5. Improvements in sedation training are imperative if conscious sedation is to emerge as the primary method alternative to general anesthesia in dental practice.
6. There is no clear guidance on conscious sedation issued by the Egyptian Dental Association or any other medical regulating agency for dental practitioners in Egypt, and this information may help health policy makers develop strategies to improve patient dental care.

## Data Availability

The datasets used and analysed during the current study available from the corresponding author on reasonable request.

## Abbreviations

AAPD: American Academy of Pediatric Dentistry
CI: Confidence interval
PV: Probability of chance value
GP: General practitioner
OR: Odds ratio
Ref: Reference

## References

[1] Caltabiano ML, Croker F, Page L (2018). Dental anxiety in patients attending a student dental clinic. BMC Oral Health..

[2] Silveira ER, Cademartori MG, Schuch HS, Armfield JA, Demarco FF (2021). Estimated prevalence of dental fear in adults: A systematic review and meta-analysis. J. Dent..

[3] Abdel Fattah MA, Barghouth MH, Wassel MO (2022). Epidemiology of dental caries in permanent dentition: Evidence from a population-based survey in Egypt. BMC Public Health..

[4] American Academy of Pediatric Dentistry. Behavior guidance for the pediatric dental patient. The Reference Manual of Pediatric Dentistry. Chicago, Ill.: American Academy of Pediatric Dentistry; 2023:359–77

[5] Mahdavi A, Fallahinejad Ghajari M, Ansari G, Shafiei L (2018). Intranasal premedication effect of dexmedetomidine versus midazolam on the behavior of 2-6-year-old uncooperative children in dental clinic. J. Dent. (Tehran)..

[6] Coté CJ, Wilson S (2019). Guidelines for monitoring and management of pediatric patients before, during, and after sedation for diagnostic and therapeutic procedures. Pediatr. Dent..

[7] Foley J (2002). The way forward for dental sedation and primary care?. Br. Dent. J..

[8] Society for the Advancement of Anaesthesia in Dentistry (UK). Standards in Conscious Sedation for Dentistry Report of an Independent Expert Working Group London: Society for the Advancement of Anaesthesia in Dentistry; 2000.

[9] American Dental Association. (2016). Guidelines for Teaching Pain Control and Sedation to Dentists and Dental Students.

[10] Krauss B, Green SM (2006). Procedural sedation and analgesia in children. Lancet.

[11] Gozal D, Gozal Y (2008). Pediatric sedation/anaesthesia outside the operating room. Curr. Opin. Anaesthesiol..

[12] Joint Commission on Accreditation of Healthcare Organizations. Comprehensive accreditation manual for hospitals. Oakbrook Terrace, IL: JCAHO;2005.

[13] Wetzel RC (2006). Do not confuse the anaesthetic with the anesthesiologist!. Anesth. Analg..

[14] Lalwani K, Michel M (2005). Pediatric sedation in North America children’s hospitals: A survey of anaesthesia providers. Paediatr. Anaesth..

[15] Wilson PH, Boyle CA, Smith BJ (2006). Conscious sedation training received by specialist registrars in restorative dentistry in the UK: A survey. Br. Dent. J..

[16] Shearer J, Wilson KE, Girdler NM (2004). A survey of the opinion of consultant anaesthetists in Scotland of sedation conducted by dentist. Br. Dent. J..

[17] Al-Shayyab MH, Ryalat S, Dar-Odeh N, Alsoleihat F (2013). Current sedation practice among general dental practitioners and dental specialists in Jordan: An example of a developing country. Ther. Clin. Risk Manag..

[18] Chadwick BL, Thompson S, Treasure ET (2006). Sedation in Wales: A questionnaire. Br. Dent. J..

[19] Whiston S, Prendergast MJ, Williams SA (1998). Sedation in primary dental care: An investigation in two districts of northern England. Br. Dent. J..

[20] Morse Z, Sano K, Fujii K, Kanri T (2004). Sedation in Japanese dental schools. Anesth. Prog..

[21] Costa PS, Valadao WJ, Costa LR (2010). Dental sedation by dentists: A view from anesthesiologists working in central Western Brazil. Anesth. Analg..

